# Magnetic Resonance-Guided Laser Interstitial Thermal Therapy for Hypothalamic Hamartoma: Surgical Approach and Treatment Outcomes

**DOI:** 10.3390/jcm11216579

**Published:** 2022-11-06

**Authors:** Yuan Yao, Xiu Wang, Wenhan Hu, Chao Zhang, Lin Sang, Zhong Zheng, Jiajie Mo, Chang Liu, Jiaji Qiu, Xiaoqiu Shao, Jianguo Zhang, Kai Zhang

**Affiliations:** 1 Department of Neurosurgery, Beijing TianTan Hospital, Capital Medical University, Beijing 100070, China; 2 Beijing Neurosurgical Institute, Capital Medical University, Beijing 100070, China; 3 Department of Neurosurgery, Beijing FengTai Hospital, Beijing 100070, China

**Keywords:** hypothalamic hamartoma, MRgLITT, gelastic seizures

## Abstract

Hypothalamic hamartoma (HH) is a rare lesion consisting of normal neurons and neuroglia arranged in an abnormal pattern which usually causes gelastic seizures (GS). Magnetic resonance-guided laser interstitial thermal therapy (MRgLITT) has been developed as a minimally invasive approach to treat HH and gradually become a first-line treatment. In total, this study enrolled 47 consecutive HH patients that underwent one round of ablation. Patients were followed for at least one year. Patients’ medical records and surgical information were carefully reviewed, and univariate analyses were performed. Of the treated patients, 72.3% remained GS-free in this study, with an overall Engel class I rate of 68.1%. Long-term postoperative complications occurred in six patients. Factors associated with GS prognosis included Delalande classification (*p* = 0.033), HH volume (*p* = 0.01), and the ablation rate of the HH body (*p* = 0.035). The disconnection rate was 0.73 ± 0.14 in the Engel class Ia group as compared to 0.62 ± 0.13 in the Engel Ib–Engel IV group (*p* = 0.046). MRgLITT represents a safe and effective surgical procedure. Patients with larger or Delalande type IV HH may require multiple rounds of ablation. In addition to assessing the degree of disconnection, ablation volume should also be carefully considered for patients undergoing this procedure.

## 1. Introduction

Hypothalamic hamartoma (HH) is a rare, non-neoplastic heterotopic lesion consisting of normal neurons and neuroglia arranged in an abnormal pattern [1]. These lesions arise in the ventral hypothalamus and can result in symptoms such as cognitive impairment, behavioral changes, precocious puberty, and seizures. Gelastic seizures are among the most characteristic manifestations of HH, and they are often resistant to pharmacologic treatment [2]. While surgery can often effectively achieve seizure control [3], the deep location of HH lesions can make it challenging to achieve full disconnection or resection. Just half of the patients who undergo open or endoscopic resection surgery achieve seizure-free status [4,5], and these procedures exhibit a high risk of postoperative complications [6].

Magnetic resonance-guided laser interstitial thermal therapy (MRgLITT) has been developed as a minimally invasive approach with the potential to treat HH [7,8], and many epilepsy centers have adopted it as a first-line treatment for HH [9]. In addition to being minimally invasive, MRgLITT also exhibits the advantage of being associated with low rates of postoperative morbidity [7,10,11]. However, there have been relatively few studies describing the surgical outcomes in patients who undergo such treatment or identifying prognostic indicators linked to operative success in a sufficiently large cohort. Accordingly, this study was developed to describe our single-center experience with this surgical approach and to identify factors associated with seizure control. In addition, the specifics of the MRgLITT surgical approach used to treat HH are described in detail. This is also the first study to report the use of the Sinovation LITT system.

## 2. Materials and Methods

### 2.1. Patients

Results from 47 patients with magnetic resonance imaging (MRI)-confirmed HH were retrospectively reviewed. All of these patients underwent MRgLITT surgery in Beijing Tiantan Hospital between August 2020 and October 2021 and had follow-up information available for a minimum of 12 months. The clinical data of each patient were reviewed, including their inpatient medical and surgical records, video-electroencephalogram (EEG) results, MRI, postoperative images, and seizure outcomes. All patients underwent preoperative 3T MRI scans (Siemens, Munich, Germany), including 3D-T1WI MPRAGE (three-dimensional T1-weighted Magnetization-Prepared Rapid Acquisition Gradient Echo), 3D-T2WI TSE (3D T2-weighted Turbo Spin Echo), 3D-T2WI FLAIR (3D T2-weighted Fluid Attenuated Inversion Recovery), TWIST (Time-resolved Angiography with Stochastic Trajectories), T2-STIR (T2-weighted Short-tau Inversion Recovery), and MRA (Magnetic Resonance Angiography). Surgical recommendations for all patients were made following a pre-surgical multidisciplinary patient management conference. The institutional review board of Beijing Tiantan Hospital, Capital Medical University approved this study.

### 2.2. Surgical Procedure

The Sinovation (Beijing, China) laser ablation system was used to treat all patients, with a Sinovation frameless surgical robot being used to assess laser catheter implantation. Following the completion of all pre-surgical examinations and evaluations, patient imaging data were imported into the SinoPlan software (v 2.1; Beijing, China) to establish an ablation plan. The implantation trajectory and ablation area were selected so as to avoid all critical structures, including the hypothalamus, internal carotid artery, fornical fibers, mammillary bodies, and mamillothalamic tract, in an effort to avoid serious complications. The T2-STIR sequence was used to discriminate the white matter fibers from the HH body in this planning protocol. The primary goal of this procedure was full disconnection between the lesion and normal brain tissue [12].

On the day of the surgical procedure, five bone markers were fixed on the patient’s skull under local anesthesia. A CT scan (0.625 mm slice thickness) was performed for robot registration. After general anesthesia was initiated, the patient’s head was fixed using an MRI-compatible head frame in the operating room. Entry point confirmation was achieved with robot assistance, and a small incision was made at the selected entry point, with a drill then being used to establish a borehole. The dura was opened through the borehole via electrocautery, and a guide bolt was firmly fixed to the skull entrance with guide rod assistance. A stylet measured to the length of the trajectory was then inserted to create an unobstructed channel for the laser catheter. Following stylet removal, the laser cannula was introduced into the channel, and the laser fiber was implanted and fixed in the cannula. Following an intraoperative MRI room safety check and the confirmation of proper laser and circulating fluid paths, the MRI device was brought into the operating room. 

The actual laser fiber position was identified through T2WI TSE scans fused with preoperative MRI images. T2-weighted Gradient Echo Pulse (GRE) sequences were also used to select four constant temperature areas (excluding the ablation region, blood vessel, and air). Thermal safety points close to important blood vessels and brain regions were defined, and a test ablation was performed at a lower energy level (3 W) to ensure that the heat source was optimally positioned. The laser power was then increased to initiate ablation. During this procedure, T2-weighted GRE scans were conducted every 3 s until the ablated area simulated by the computer was satisfactory or a warning temperature was achieved. If the targeted ablation region was relatively large, after the temperature had recovered to the reference temperature, the laser fiber was retracted and fixed again as appropriate under aseptic conditions, after which the above steps were repeated.

Following ablation, T2WI TSE and enhanced T1WI scans were used to confirm the actual ablation range and volume. Postoperative hemorrhage was excluded through a CT scan. Hormone measurements were performed on the day immediately following surgery, prior to hospital discharge, and monthly thereafter until they had recovered to normal levels. For patients with hormone deficiency, replacement therapy was applied immediately until hormone levels return to normal.

### 2.3. Surgical Data Collection

**Figure 1:**
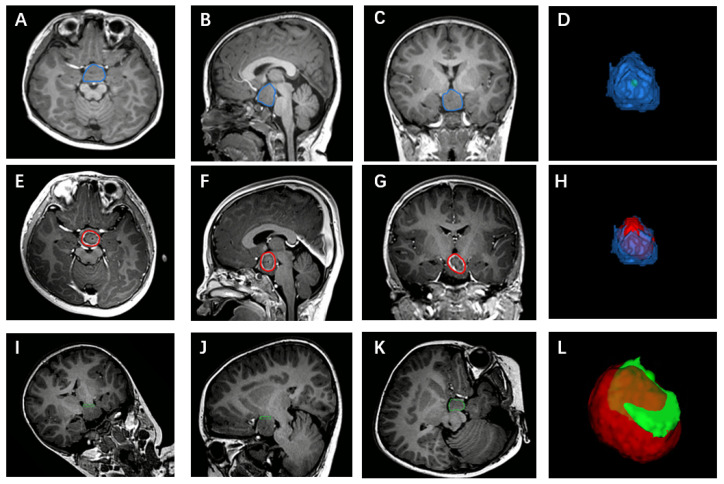
(**A**–**D**) The construction of the HH body. (**D**): The constructed 3D model of HH body. (**E**–**H**) The construction of the ablation area. (**H**): The 3D model of ablation area and HH body. (**I**–**L**) The construction of the border between HH and normal brain tissue. (**L**): The 3D model of the border and ablation area. The circled area was manually outlined to constructed 3D model.

HH morphological characteristics and surgical ablation-related information were collected for all patients. HH Delalande classification [13] and the laterality were confirmed by two senior epilepsy neurosurgeons based upon MR images. When HH lesions exhibited bilateral attachment to the third ventricle, decisions were with reference to EEG data. HH volumes were calculated using the planning system software (SinoPlan version 2.1). The lesion margin was manually outlined slice-by-slice in 3D-T1WI MPRAGE images [repetition time (TR) = 2200 ms, echo time (TE) = 2.26 ms, slice thickness = 1 mm, voxel size = 1 mm × 1 mm × 1 mm], with the resultant images then being combined to generate a 3D hamartoma model for volume calculation [14]. Postoperative enhanced T1 images obtained immediately after the ablation (repetition time (TR) = 2300 ms, echo time (TE) = 2.3 ms, slice thickness = 1 mm, voxel size = 1 mm × 1 mm × 1 mm) and preoperative MRI scans were fused to construct a 3D model of the actual ablation area, with the ablation rate being defined by the percent of HH body ablated. A 3D model of the connected region was also generated as follows: Initially, T1 images were rotated around to Z-axis to ensure that the HH border was parallel to the X-axis, with this border being defined by the intensity or angle differences of the third ventricle wall [12]. Then, three consecutive slices from the border to the HH body were selected and used to outline the edges of the connected part, thereby yielding a 3D model. The volume overlapping between this connected region and the actual ablation area was then calculated to establish the disconnection rate (Figure 1).

### 2.4. Statistical Analysis

The association between clinical variables and patient outcomes was assessed through univariate analyses via two-sample t-tests, χ^2^ tests, and Fisher’s exact test. SPSS 26.0 was used for all analyses, with *p* < 0.05 as the significance threshold. Normally and non-normally distributed data are respectively reported as the mean ± standard deviation or median (range).

## 3. Results

### 3.1. Patient Information

A total of 47 patients underwent LITT surgeries from August 2020 to July 2021 and each patient underwent only one surgery. All patients finished at least 12 months of follow-up, with a median follow-up time of 15.5 months (range: 12–21 months). Demographic details and univariate analyses for these patients are provided in Table 1. These patients exhibited respective median ages at surgery and seizure onset of five years (range: 2–33 years) and one year (range: 0–12 years). Six patients had a history of other operations, including resection, gamma knife, and radiofrequency thermocoagulation procedures. The median duration of postoperative hospitalization was four days (range: 2–7 days), and no patients were admitted to the ICU after surgery. None of these variables were associated with patient prognosis.

### 3.2. Predictors of Patient Prognosis

Of the 47 patients in our cohort, nine experienced only GS, while 38 experienced GS and other seizure types including focal impaired awareness seizure, focal motor seizure, and focal to bilateral tonic–clonic seizure. There were no differences in posting these two groups of patients (*p* = 0.211). Prior to surgery, 13 patients were diagnosed with precocious puberty, which was unrelated to patient prognosis. Seizure outcomes were also unrelated to HH laterality (*p* = 0.927), nor were they impacted by whether or not the attachment was unilateral or bilateral (*p* = 0.125). Significant differences in GS outcomes were evident when comparing the four Delalande classifications (*p* = 0.033). Specifically, Type I patients achieved the best outcome with a 100% GS-free rate, whereas type IV patients achieved the worst outcomes with a GS-free rate of just 40%. The respective GS-free rates for Type II and III patients were 86.7% and 66.7%. Due to the disruption of the initial structure during prior surgical procedures, two patients were not classified. For further details, see Table 2.

Ipsilateral and contralateral laser fiber implantation procedures were respectively performed in 30 and 17 patients, and there were no significant differences in prognosis between these groups. Just six patients in this study cohort were implanted with 2 laser fibers. With respect to the trajectory path, 36 and nine patients respectively underwent anterior and posterior implantation, while two underwent implantation via both directions. These factors were unrelated to postoperative GS-free rates (Table 3).

Ablation rates in the GS-free group (0.69 ± 0.15) were higher than those in the group of patients that continued to experience GS (0.56 ± 0.16, *p* = 0.035), although there was no difference in disconnection rate between these groups (*p* = 0.43). Further analyses suggested that there were differences in disconnection rates between Engel Ia patients (0.53 ± 0.14) and Engel Ib–Engel IV patients (0.42 ± 0.13, *p* = 0.046). Larger HH lesions also exhibited a worse prognosis relative to smaller lesions (*p* = 0.01).

### 3.3. Seizure Outcomes

Overall, 72.3% of patients (34/47) remained GS-free as of the most recent follow-up. The Engel classifications for these patients were as follows: 32 Engel I (68.1%), six Engel II (12.8%), four Engel III (8.5%), and five Engel IV (10.6%). Of these patients, 38 had experienced other seizure types besides GS, while 30 (78.9%) free of these seizure types and 8 (21.1%) continued to experience non-GS attacks after surgery. Five patients experienced memory disturbances immediately after the procedure, four recovered within a few weeks, while one patient continued to experience memory dysfunction as of most recent follow-up. Two patients experienced postoperative obesity as of most recent follow-up (Table 4). Postoperative hormone levels were also analyzed in these patients (Table 5), including thyroxine (T4), triiodothyronine (T3), free thyroxine (FT4), free triiodothyronine (FT3), thyroid stimulating hormone (TSH), prolactin (PRL), cortisol, growth hormone (GH), Luteinizing hormone (LH), follicle-stimulating hormone (FSH), and estradiol (E2) levels. Patients that exhibited preoperative hormone abnormalities were not included in this analysis. TSH and cortisol deficiencies were observed in 14 (14/35, 40%) and 34 (34/44, 77.3%) patients, respectively, within one week after the surgery. Most of these patients exhibited normal hormone levels as of most recent follow-up, with the exception of three patients that still exhibited cortisol deficiencies. The median time for TSH recovery and cortisol recovery was three (2–97) days and 83 (3–188) days, respectively.

## 4. Discussion

In summary, clinical data from 47 HH patients were herein collected and analyzed to better understand outcomes associated with the LITT procedure and to define predictors of patient prognosis. All patients were followed for at least 12 months. By presenting these clinical data from our center, we hope to provide a valuable reference to support the widespread application of LITT as a means of treating HH. We additionally calculated volume-related parameters and examined their correlations with patient prognosis through the construction of 3D models, providing further insight regarding the HH procedure and assisting in the ablation planning process. GS incidence served as the primary prognostic readout in our study, given that there have been reports that HH lesions are the origin of GS based on stereoelectroencephalography [15,16] and neuropathological [1] evidence. Rates of other seizure types associated with discharges from affecting cortical regions were also evaluated in these patients.

Several approaches to HH treatment have been defined to date. The first successful surgical resection of an HH lesion was reported in 1967 [17], but open surgical approaches have largely been replaced by minimally invasive procedures given that they only yield a ~50% seizure-free rate and are associated with a high risk of hypothalamus damage-related endocrine dysfunction, weight gain, and memory deficits [4,13,18]. While endoscopic resection or disconnection can achieve better seizure-related prognostic outcomes, it is also associated with high complication rates and is not suitable for treating some patients [5,19]. Stereotactic radiofrequency thermocoagulation (SRT) has been reported as an effective and minimally invasive treatment for HH [20,21], but it can achieve a much smaller ablation volume than the LITT procedure and does not incorporate any thermal monitoring procedures to permit the intraoperative assessment of the true ablation range. stereotactic radiosurgery (SRS) or gamma knife surgery (GKS) are noninvasive treatment strategies that exhibit much lower complication rates [22]. However, because of its delayed therapeutic effect following an increase in seizures, their use in children with progressive epileptic encephalopathy is not recommended [23]. Focused ultrasound (FUS) thermoablation is a recently described form of novel noninvasive treatment for HH, but appropriate efficacy and safety validation for this technique remains to be conducted [24]. Overall, the MRgLITT approach is both safe and effective as a means of treating HH.

### 4.1. Seizure Outcomes

MRgLITT is growing in popularity as a treatment for HH, offering immediate postoperative efficacy while causing minimal injury to surrounding structures and enabling shorter postoperative hospitalization [25]. In 2012, Curry et al. first reported outcomes from five epilepsy patients treated via MRgLITT and thereby demonstrated the value of applying this technique [26]. Since that initial description, the largest published HH study cohort included 71 patients that underwent LITT treatment, achieving a 93% GS-free rate, with 23% of patients requiring more than one ablation procedure [7]. Relative to that previous report, the GS-free rate in our cohort was lower as none of patients underwent two or more ablation procedures. This emphasizes the importance of performing multiple ablations in some patients. HH classification is another factor likely associated with these differences in patient prognosis. The cohort published by Curry et al. included nine Delalande type 4 patients, accounting for 12.7% of their overall cohort, which is lower than the 21.3% in our cohort. To date, only data for a single cohort with a >6-month follow-up duration and more than 20 cases has been published, as reported by Gadgil et al. [8]. These authors reported a GS-free rate of 81%, which was also higher than ours, likely for the same reasons discussed above. However, in their study, the rate of patients that achieved significant improvements including GS-free patients and patients with a notable decrease in GS incidence was 89.6%, in line with the 87.2% rate in our cohort. In addition, our Engel I rate (68.1%) was similar to that in the study published by Gadgil et al. (69%). In another retrospective study of 18 patients with a follow-up duration of at least 6 months, Xu et al. [27] reported an 80% postoperative GS-free rate, a 72.2% Engel class I rate, and a need for multiple ablations in 16.7% of patients. However, just one patient in their cohort was Delalande type 4. Seizure-free rates for other seizure types were also reported in these two studies as being 68.2% and 56%, respectively, with these rates being lower than our study. One recent meta-analysis related to LITT for HH patients reported an overall seizure-free rate of 67% (95% CI 0.57% to 0.76%) [28], similar to our results.

With respect to postoperative complications, one patient experienced long-term memory deficits. Weight gain occurred in two patients. three patients experienced endocrine dysfunction. Long-term endocrine dysfunction has previously been reported in patients undergoing LITT treatment for HH in the form of hypothyroidism [27]. In the present cohort, both affected patients exhibited cortisol deficiencies, and a review of the results of prior hormone tests revealed that changes in both TSH and cortisol levels were common within one week after the LITT procedure, although most of these levels returned to normal over the following weeks and months, with cortisol exhibiting a longer recovery time. This emphasizes the importance of routinely assessing postoperative hormone levels in patients that undergo LITT treatment for HH. The observed cases of cortisol deficiency may be attributable to the impact of the ablation on the neuroendocrine hypothalamic nuclei, pituitary stalk, or gland [29]. Overall, the complications observed in our patient cohort are in line with prior reports and attest to the safety of the LITT procedure.

### 4.2. Surgical Strategy

**Figure 2:**
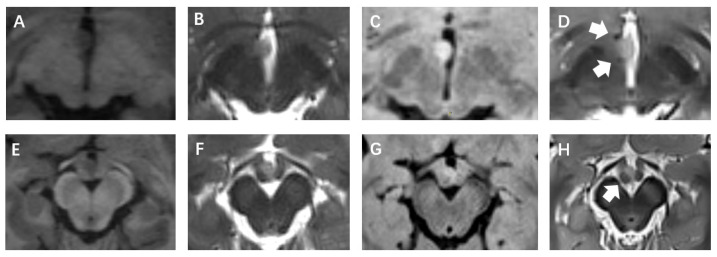
(**A**,**E**) T1-MPRAGE. (**B**,**F**) 3D-T2WI TSE. (**C**,**G**) 3D-T2WI FLAIR. (**D**,**H**) T2-STIR. The white arrows in (**D**) point to the fornix and mammillothalamic tract, respectively. In (**H**), it points to the mammillary body.

The primary goal of surgical ablation was to disconnect the HH from normal brain tissue via the destruction of the HH basement layer, thereby achieving satisfactory seizure control. Prior studies have reported on the importance of disconnection in HH treatment [21,30]. During our trajectory planning process, entry point design was separated into two categories: anterior and posterior approaches. The anterior path entailed the use of an entry point in the frontal lobe and was generally considered to be safer than the posterior approach as the resultant trajectory was shorter and crossed fewer important structures. As such, the anterior path was usually the first choice when designing. Posteriorly growing HH lesions in the sagittal plane necessitated the use of a posterior approach employing an entry point generally set at the postcentral gyrus in the parietal lobe in order to achieve sufficient ablation. However, this approach necessitated a longer path and required the crossing of central region, the posterior limb of internal capsule and the thalamus. Computers were used to estimate the maximum ablation range achievable through adjustments to power and time in the surgical planning software, with an estimated range being selected to cover as much of the HH border as possible and to thereby determine an appropriate target point. It is critical that several crucial structures be protected when selecting the target ablation area. Care must be taken to avoid the mammillary body, mammillothalamic tract, and fornix in order to prevent any short- or long-term memory deficits. Our center utilized T2-STIR sequences to differentiate HH lesions from the mammillary body and mammillothalamic tract during ablation planning. Because it has higher contrast between HH and surrounding structures than other sequences (Figure 2). In order to minimize the incidence of endocrine complications, the hypothalamus must be avoided to the greatest extent possible. The optic tract and blood vessels must also be protected to prevent hemorrhage or damage to the visual field. To ensure greater accuracy during catheter implantation, the path should also be perpendicular to the surface of the skull and avoid the ventricle.

The number of fibers is primarily determined based on HH lesion morphological characteristics. The use of one laser fiber was standard except in the following situations: (1) when HH lesion attachment to both high and low areas in the third ventricle is evident, in which an additional fiber was considered to achieve full ablation of the connected area; or (2) when cystic changes were evident in the HH lesion, as cyst fluid exhibits lower conductivity relative to the HH body, and another fiber may thus be required to separately ablate the target lesion.

**Figure 3:**
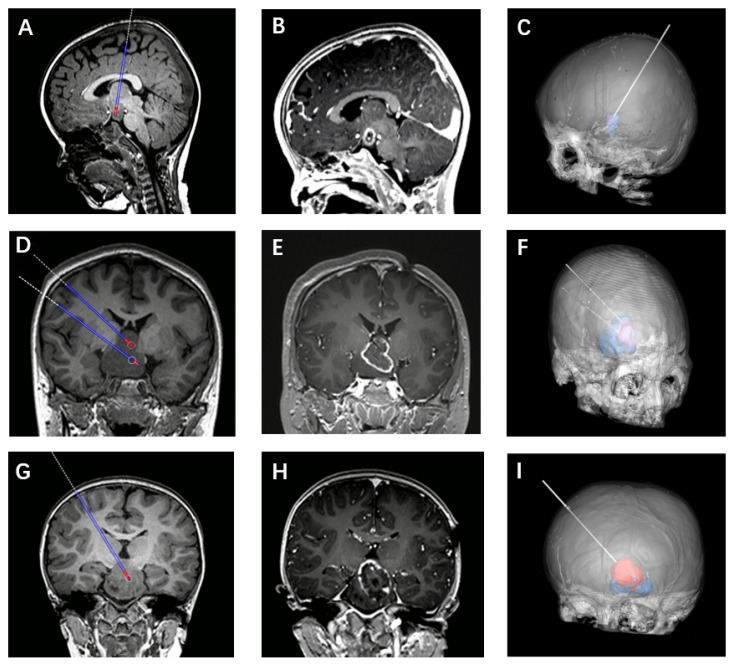
(**A**–**C**) Showing the posterior approach of laser implantation. (**D**–**F**) Two laser fibers were used in one patient. (**G**–**I**) The laser fiber was implanted contralaterally.4.3. Factors associated with patient prognosis.

The laterality of laser fiber implantation was dependent on the condition of attachment, with ipsilateral placement being appropriate for patients with HH lesions exhibiting a narrower attachment surface so as to avoid interfering with the contralateral hypothalamic structures. In cases of HH lesions exhibiting broad attachment, contralateral placement is better suited to achieving complete disconnection (Figure 3).

In our analyses, the prognosis of the patients with Delalande type 4 HH differed significantly from that of other patients. GS-free rates for type 1 and type 2 patients were notably better (100% and 86.7%, respectively), whereas type 4 patients exhibited the worst outcomes. This is likely attributable to the larger HH body and wider connection in type 4 HH. While a prior study did not report differences among different HH classifications, type 4 patients nonetheless exhibited the worst outcomes [8]. Volume analyses were also conducted in this study, revealing that both a larger HH volume and a lower HH body ablation rate as calculated after surgery were associated with poorer GS prognosis. This is likely because achieving 100% disconnection between larger HH lesions and the normal brain tissue is more challenging owing to the irregular morphological contact surface. Even so, reducing the HH body volume in these cases can reduce epileptogenic discharges and thereby help achieve seizure control. This finding emphasizes the importance of considering HH volume both before and during the ablation independent of disconnection. No differences in disconnection rate were observed among different GS prognoses, although significant differences were evident between the Engel class Ia and Engel class Ib–Engel IV groups. Specifically, disconnection rates were higher in the former group, validating our surgical strategy. The only prior study to have conducted volumetric analyses was published by Gadgil et al., who reported an association between the GS-free rate and the percent of residual hamartoma, in line with our results. However, they reported a distinct relationship between Delalande classification and prognosis from our results, underscoring the need for further validation studies with a larger sample size.

## 5. Conclusions

The results of this study indicate that LITT is a safe and effective approach to treating HH patients. In our experience, this minimally invasive procedure is associated with satisfactory seizure outcomes relative to previously utilize open and endoscopic surgical approaches while causing fewer complications. Factors associated with the prognosis of patients undergoing this procedure included HH morphological classification, ablation rate of the HH body, HH volume, and disconnection rate, providing further guidance for ablation planning efforts.

## Figures and Tables

**Table 1:** General demographics.

General Demographics	Free of GS (N = 31)	Continued GS (N = 16)	Total (N = 47)	*p*
Gender				0.761
male	24	13	37	
female	7	3	10	
Age at surgery (years)	5.0 (2–30)	3.5 (2–33)	5 (2–33)	0.466
Age of seizure onset (years)	1.3 (0–11)	0.4 (0–12)	1 (0–12)	0.086
Operation history	6	0	6	
GKS			1	
Resection			4	
SRT			1	

GS: gelastic seizure. GKS: Gamma Knife surgery. SRT: Stereotactic radiofrequency thermocoagulation.

**Table 2:** HH information.

HH Information		Free of GS (N = 34)	Continued GS (N = 13)	Total (N = 47)	*p*
Seizure types	Only GS	5	4	9	0.211
	GS with other seizure types	29	9	38	
Delalande classification	I	5	0	5	0.033 *
	II	13	2	15	
	III	10	5	15	
	IV	4	6	10	
	Unrecognizable	2		2	
Laterality of HH	Left	14	7	21	0.927
	Right	17	9	26	
Attachment condition	Unilateral Bilateral	17 14	5 11	22 25	0.125
Precocious puberty	with	7	6	13	0.279
	without	24	10	34	

HH: hypothalamic hamartoma. *: *p*-values < 0.05

**Table 3:** Surgery information.

Surgery Information		Free of GS	Continued GS	Total	*p*
Laterality of laser fiber implantation	Ipsilateral	23	7	30	0.094
	Contralateral	9	8	17	
Number of laser fiber	1	27	14	41	0.969
	2	4	2	6	
Trajectory path	Anterior	23	13	36	0.860
	Posterior	6	3	9	
	Both	2	0	2	
HH volume (mm^3^)		886.45 (215.16–6152.84)	4791.90 (327.27–19,667.10)	1310.45 (215.16–19,667.10)	0.01 *
Ablation rate		0.69 ± 0.15	0.56 ± 0.16	0.64 ± 0.18	0.035 *
Disconnection rate		0.71 ± 0.16	0.66 ± 0.13	0.49 ± 0.15	0.43

*: *p*-values < 0.05.

**Table 4:** Seizure outcome.

Seizure Outcome		
Engel classification	I	32 (68.1%)
	II	6 (12.8%)
	III	4 (8.5%)
	IV	5 (10.6%)
Gelastic seizures	Free of GS	34 (72.3%)
	Reduction of GS > 80%	7 (14.9%)
	Continued GS	13 (27.7%)
	Total	47
Other seizure types	Free of other seizure types	30 (78.9%)
	Continued Other seizure types	8 (21.1%)
	Total	38
Long-term complications	Memory deficit	1
	Weight gain	2
	Endocrine	3

**Table 5:** Hormone.

Hormone	Postoperative Abnormality (%)	Full Recovery (%)	Recovery Time (Days)
T3	3/35 (8.6)	3/3 (100)	2,4,4
T4	1/37 (2.7)	1/1 (100)	4
FT4	4/34 (11.8)	4/4 (100)	3,4,4,5
FT3	0/34 (0)	-	-
TSH	14/35 (40)	14/14 (100)	3 (2–97)
PRL	3/31 (9.7)	3/3 (100)	3,4,4
GH	4/40 (10)	4/4 (100)	5,4,4,3
Cortisol	34/44 (77.3)	31/34 (91.2)	83 (3–188)
LH	3/25 (12)	3/3 (100)	3,3,4
FSH	1/37 (2.7)	1/1 (100)	7
E2	1/43 (2.3)	1/1 (100)	3

Thyroxine (T4), triiodothyronine (T3), free thyroxine (FT4), free triiodothyronine (FT3), Thyroid stimulating hormone (TSH), prolactin (PRL), growth hormone (GH), Luteinizing hormone (LH), Follicle stimulating hormone (FSH), Estradiol (E2).

## Data Availability

The data presented in this study are available on request from the corresponding author. The data are not publicly available due to privacy regulations for patients.
